# Supplementary material for: Discovery of giant unit-cell super-structure in the infinite-layer nickelate PrNiO2+x
Source: Commun Mater. 2025 Jan 7;6(1):3. doi: 10.1038/s43246-024-00729-4 (PMC11703755; doi:10.1038/s43246-024-00729-4)
Supplement: Supplementary file 2 — Supplementary Information [file 43246_2024_729_MOESM2_ESM.pdf]

Discovery of Giant Unit-Cell Super-Structure in the Infinite-Layer Nickelate $PrNiO_{2+x}$

Jens Oppliger,[1,*] Julia Küspert,[1] Ann-Christin Dippel,[2] Martin von Zimmermann,[2]
Olof Gutowski,[2] Xiaolin Ren,[3] Xingjiang Zhou,[3] Zhihai Zhu,[3] Ruggero Frison,[1]
Qisi Wang,[4] Leonardo Martinelli,[1] Izabela Biało,[1] and Johan Chang[1,†]

[1] *Physik-Institut, Universität Zürich, Winterthurerstrasse 190, CH-8057 Zürich, Switzerland*
[2] *Deutsches Elektronen-Synchrotron DESY, Notkestraße 85, 22607 Hamburg, Germany.*
[3] *Beijing National Laboratory for Condensed Matter Physics, Institute of Physics, Chinese Academy of Sciences, Beijing 100190, China*
[4] *Department of Physics, The Chinese University of Hong Kong, Shatin, Hong Kong, China*

SUPPLEMENTARY INFORMATION

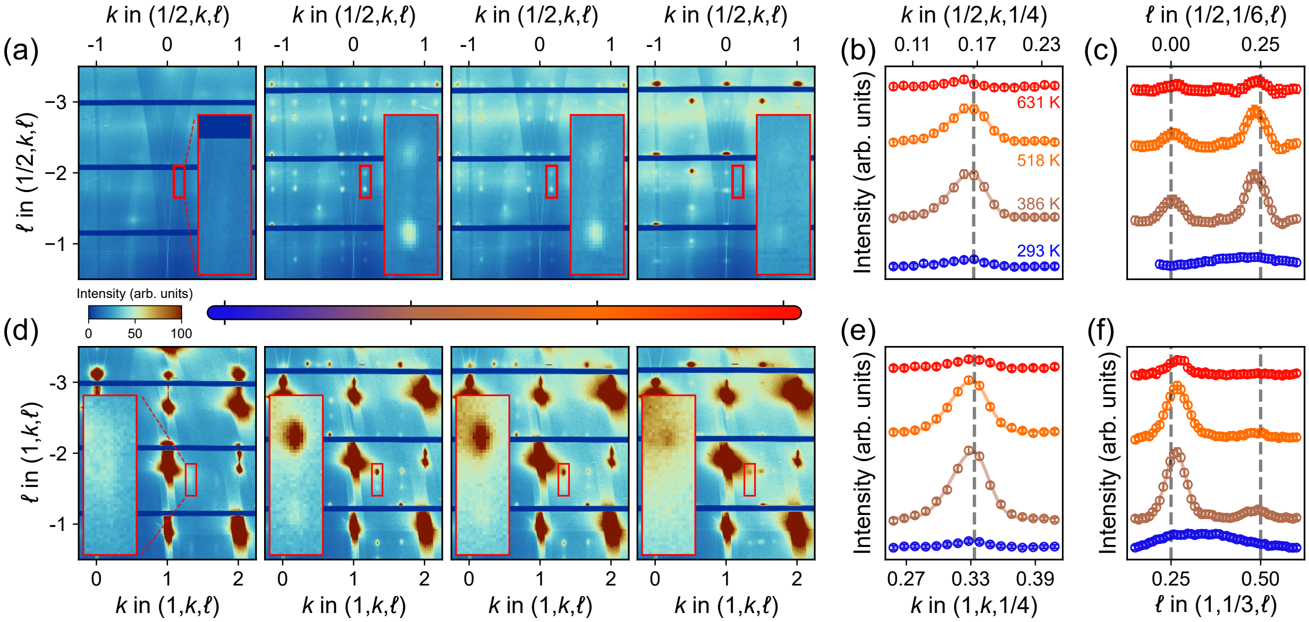

SUPPLEMENTARY FIGURE 1. **Thermally-induced superlattice structure in a $PrNiO_{2+x}$ thin film.** (a,d) Diffraction intensities (displayed with a linear false color scale) in the $(1/2, k, \ell)$ and $(1, k, \ell)$ scattering planes as a function of temperature. The most intense peaks stem from fundamental Bragg peaks of the $SrTiO_3$ substrate and the $PrNiO_{2+x}$ thin film. Selected superlattice peaks are highlighted by red rectangular boxes. (b,c,e,f) One-dimensional k (in-plane) and ℓ (out-of-plane) scans through the superlattice reflections for temperatures as indicated. Solid lines are Gaussian profiled fits with a sloping background. Error bars reflect counting statistics.

* jens.oppliger@physik.uzh.ch

† johan.chang@physik.uzh.ch

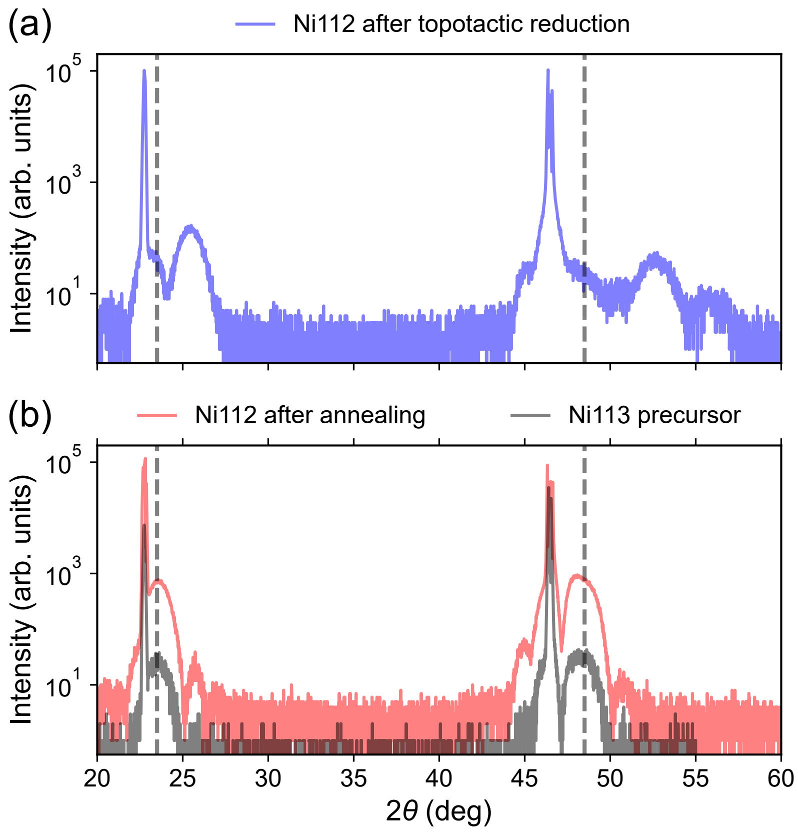

SUPPLEMENTARY FIGURE 2. **Room temperature $\theta - 2\theta$ scans of thin films.** (a) Infinite-layer phase $PrNiO_2$ (Ni112) obtained after topotactic reduction of the perovskite precursor $PrNiO_3$ (Ni113) with crystalline STO capping layer. (b) Comparison of the Ni112 thin film after annealing at high temperatures with the original Ni113 phase. The scans were performed using a SmartLab x-ray diffractometer with a Cu Kα source.
